# The Therapeutic Effect of GPR81 in Autoimmune Hepatitis and Hepatocellular Carcinoma via Regulating the Immune Response

**DOI:** 10.3390/ijms26136308

**Published:** 2025-06-30

**Authors:** Yongmei Wu, Wenqian Song, Xuxian Wu, Jing He, Min Su, Rong Hu, Youbo Zhao

**Affiliations:** Department of Human Histology and Embryology, Center for Tissues Stem Cell and Translational Medicine Research, Key Laboratory of Regenerative Medicine in Guizhou Province, Guizhou Medical University, Guiyang 550025, China; wuyongmei1990@sina.com (Y.W.); song_wenqian06@163.com (W.S.); wuxuxiangmc@sohu.com (X.W.); hejingmed@163.com (J.H.); sumin@gmc.edu.cn (M.S.)

**Keywords:** GPR81, 3,5-DHBA, 3-OBA, autoimmune hepatitis, immune microenvironment, hepatocellular carcinoma

## Abstract

Autoimmune hepatitis (AIH) is linked to an increased risk of hepatocellular carcinoma (HCC). However, the precise connection between the two remains unclear. GPR81, a G-protein-coupled receptor located on the membranes of various cell types, plays a role in numerous physiological processes. We established an AIH animal model and activated GPR81 using the agonist 3,5-dihydroxybenzoic acid (3,5-DHBA). Additionally, the effect of GPR81 inhibition on tumor and immune cell dynamics was examined using the HepG2, Hep3B, and Hepa1-6 cell lines with the antagonist 3-hydroxybutyric acid (3-OBA). Our results demonstrated that 3,5-DHBA treatment reduced T cell and pro-inflammatory cytokine secretion, while MDSC secretion increased, inhibiting Concanavalin A (Con A)-induced AIH. The inhibition of GPR81 by 3-OBA suppressed HCC cell proliferation and invasion, reduced tumor volume and weight, and downregulated PD-L1 expression. Furthermore, CTL and DC activity in the spleen and tumors increased, while MDSC activity decreased. This study confirms that GPR81 plays an important role in both inflammation and tumorigenesis, suggesting that GPR81 may serve as a bridge in the transformation of inflammation into cancer. Modulating GPR81 activity may provide a novel therapeutic strategy for hepatitis and cancer.

## 1. Introduction

Inflammatory cancer transformation plays a critical role in the development of HCC, driven by sustained cellular proliferation within an inflammatory microenvironment composed of inflammatory cells, growth factors, activated stroma, and DNA-damaging factors [1]. This inflammatory state contributes to cirrhosis and hepatocyte dysfunction, increasing cancer risk [2]. The hyperactivation of CD4^+^ T cells [3], cytotoxic T lymphocytes (CTLs) [4], dendritic cells (DCs) [5], and M1 macrophages [6] is central to this process. These immune cells secrete pro-inflammatory factors like IFN-γ [7], TGF-β [8], IL-17 [9], and granzyme B [10], further promoting the inflammatory environment. Autoimmune liver diseases (AILDs), including AIH, primary biliary cholangitis (PBC), and primary sclerosing cholangitis (PSC) [11], are characterized by immune-mediated liver injury, leading to fibrosis, cirrhosis, and functional impairment [12]. Despite initial treatment with glucocorticosteroids or azathioprine, the lack of response predictors leads to relapses and an increased risk of hepatic fibrosis [13]. Notably, one-third of AIH patients already have cirrhosis at symptom onset, and those with acute symptoms often exhibit advanced fibrosis or sclerosis on liver biopsy [14]. Current treatments primarily rely on immunosuppressive drugs, which are associated with varying side effects and can significantly impact quality of life. These limitations underscore the pressing need for the development of new, more effective therapeutic strategies.

GPR81, also known as HCAR1, is a G-protein-coupled receptor (GPCR) located on the cell membrane [15]. Its activation by lactate influences various biological processes, including energy homeostasis, memory formation, wound healing, ischemic injury, and cancer progression [16]. Lactate regulates TLR-induced NLRP3 inflammasome activation and limits inflammasome activation and organ damage through GPR81 [17]. Inflammation triggers lactate release from neutrophils in the bone marrow, which binds to GPR81 on endothelial cells, increasing permeability and promoting neutrophil recruitment by releasing G-CSF, CXCL1, and CXCL2 [18]. During labor, lactate affects the uterine inflammatory response via GPR81 [19], and in mice, it helps to maintain intestinal homeostasis and protects against colitis [20]. However, excessive lactate can stimulate serum amyloid A (SAA) production through the GPR81–NFκB axis, exacerbating Th1-driven inflammation [21]. Lactate also modulates immune cells like MDSCs, Tregs, CD4^+^ and CD8^+^ T cells, and NK cells, influencing their activity, proliferation, and effector functions [22]. In plasmacytoid DCs, lactate activates GPR81, mobilizing intracellular Ca_2_^+^ and regulating IFN-α production [23]. In macrophages, GPR81 modulates NF-kB and YAP via AMPK and LATS, reducing inflammatory factor release after LPS exposure [24]. These findings suggest GPR81’s key role in inflammation, but its specific function and mechanisms in AIH remain unclear.

GPR81 plays a central role in various tumors, with the tumor microenvironment (TME) presenting high lactate levels. Lactate binds to GPR81, which is highly expressed in cancer cells such as those in cervical [25], breast [26], lung [27], and pancreatic cancers [28], enhancing cell survival by promoting energy and lipid metabolism and drug resistance [29]. Inhibiting HCAR1/MCT1 to block lactate uptake activates AMPK, downregulating SCD1 and promoting tumor ferroptosis [30]. Depleting adipose-specific GPR81, similarly to global GPR81 deficiency, alleviates lactate-induced fat and muscle atrophy in male mice, delaying cancer cachexia onset [31]. GPR81 also enhances PD-L1 expression in tumor cells, suppressing immune responses [32]. Lactate regulates MDSC activation through the GPR81/mTOR/HIF-1α/STAT3 pathway, and blocking lactate production in tumor cells restores anti-tumor T cell responses [28]. The in vivo reduction in lactate levels or inhibition of GPR81 signaling decreases extracellular vesicle HMGB1 levels and improves survival outcomes in sepsis patients [33]. Our research hypothesis is that the activation of GPR81 modulates the immune microenvironment in HCC and AIH, potentially influencing disease progression. This hypothesis is based on the role of GPR81 in inflammation and cancer, particularly its effects on immune cells and inflammatory pathways.

By examining GPR81’s role in AIH, we aim to explore its potential as a therapeutic target for regulating inflammation and HCC. Our research demonstrates that GPR81 activation modulates immune cells, influencing the immune microenvironment and reducing inflammation in hepatitis. In contrast, GPR81 inhibition mitigates the malignant features of HCC and slows its progression in vivo, based on a mouse model. This study introduces the concept that lactate-induced activation of GPR81 could be a key modulator of the immune microenvironment in liver diseases.

## 2. Results

### 2.1. GPR81 Agonist 3,5-DHBA Effectively Alleviates Con A-Induced AIH

**Figure 1:**
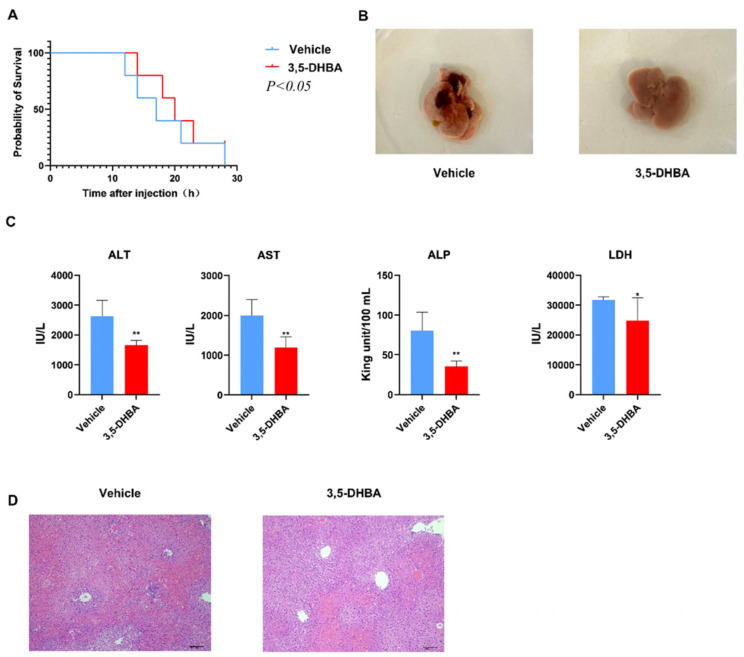
Treatment with GPR81 agonist 3,5-DHBA ameliorates Con A-induced AIH. (**A**) Survival curves of the vehicle and 3,5-DHBA groups. The statistical analysis was conducted by Logrank test (*n* = 10). Members of the 3,5-DHBA group were injected intraperitoneally with 10 mg/kg 3,5-DHBA and the NC group were injected with an equal volume of normal saline at the same time point before Con A injection, and mice were euthanized 12 h after Con A administration. (**B**) Representative macroscopic image of the liver. (**C**) ALT, AST, LDH, and ALP levels in serum. Data are presented as mean ± SD. * *p* < 0.05, ** *p* < 0.01 (Student’s *t*-test, *n* = 6 mice per group). (**D**) Representative images of H&E staining liver (scale bar 100 μm).

To investigate the protective effect of GPR81 in AIH, we used 3,5-DHBA, an agonist of the lactate receptor GPR81. First, an AIH model was established by administering a high dose of Con A (20 mg/kg) to mice. Prior to the Con A injection, mice were intraperitoneally treated with 3,5-DHBA (10 mg/kg) or saline. Survival curves indicated that 3,5-DHBA treatment significantly improved survival (Figure 1A). Due to the severe effects of the high dose of Con A, we lowered the dose to 10 mg/kg for subsequent experiments. After 12 h, mice were euthanized, and liver macroscopic observations revealed increased liver size and tense surface in the vehicle group, with dark red hyperemia in some areas. In contrast, 3,5-DHBA treatment alleviated liver hyperemia, suggesting reduced liver inflammation (Figure 1B). Serum ALT and AST levels, markers of liver injury, were significantly lower in the 3,5-DHBA group. Additionally, the levels of LDH and ALP, key indicators of acute liver damage, were markedly reduced in the 3,5-DHBA group compared to the vehicle group.

These findings suggest that 3,5-DHBA provided partial relief from acute AIH (Figure 1C). To further assess liver condition, we performed H&E staining on liver tissues. The vehicle group showed deep staining, disordered hepatocyte arrangement, ruptured hepatocytes, and significant inflammatory cell infiltration. In contrast, the 3,5-DHBA group exhibited well-arranged hepatocytes with clear boundaries, reduced necrosis, and less inflammatory infiltration (Figure 1D). These results indicate that GPR81 activation offers protective effects against AIH.

### 2.2. Pharmacological Activation of GPR81 Inhibits T Cell Activation In Vivo

**Figure 2:**
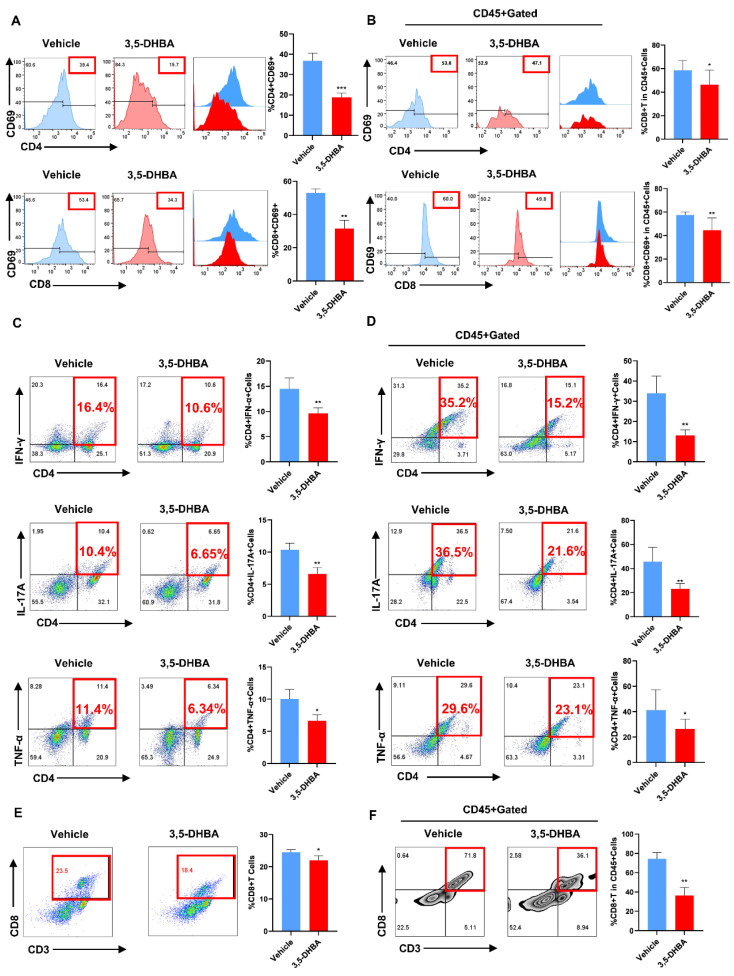
Pharmacological activation of GPR81 reduces the secretion and activity of T cells. After 12 h modeling, liver and spleen mononuclear cells were harvested and stained. (**A**,**B**) Flow cytometry was applied to analyze the expressions of CD4^+^CD69 and CD8^+^CD69 in spleen and liver. (**C**,**D**) CD4^+^IFNγ, IL-17A and TNF-ɑ expression in the spleen and liver. (**E**,**F**) CTL (CD3^+^ CD8^+^) expression in spleen and liver. Data are presented as mean ± SD. * *p* < 0.05, ** *p*< 0.01, *** *p* < 0.001 (Student’s *t*-test, *n* = 6 mice per group).

Con A-induced AIH is primarily driven by T lymphocyte activation, leading to acute liver injury. To investigate if GPR81 activation inhibits T cell activation, we collected liver and spleen tissues from the vehicle and 3,5-DHBA groups and then prepared single-cell suspensions for flow cytometry. The activation of T cells was assessed by measuring CD4^+^CD69 and CD8^+^CD69 markers. In both tissues, 3,5-DHBA significantly reduced the percentage of activated CD4^+^ and CD8^+^ T cells (Figure 2A,B). We also examined the expression of CD4^+^IFN-γ, CD4^+^IL-17A, and CD4^+^TNF-α in the liver and spleen, observing a marked decrease in the 3,5-DHBA group (Figure 2C,D). Additionally, GPR81 activation reduced CTL expression in both organs (Figure 2E,F). These results suggest that pharmacological activation of GPR81 suppresses T cell activation in mice.

### 2.3. Pharmacological Activation of GPR81 Reduces the Secretion of DCs and Promotes the Release of MDSCs to Enhance Immune Regulatory Capabilities

**Figure 3:**
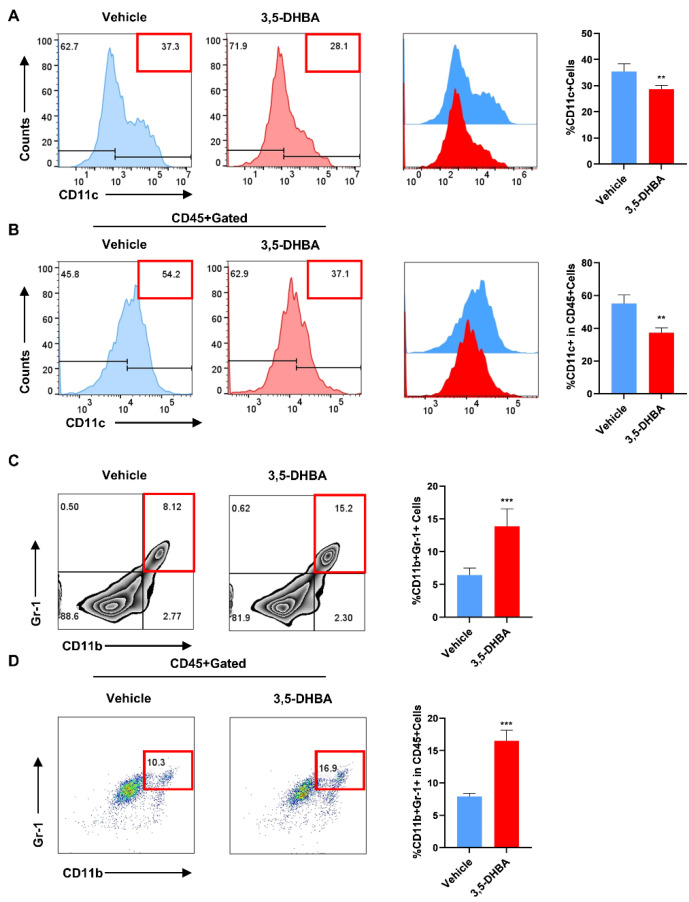
Pharmacological activation of GPR81 reduces the secretion of DCs and increases the frequency of MDSCs. (**A**) DCs (CD11c^+^) in the spleen. (**B**) DCs (CD11c^+^) in the liver. (**C**) MDSCs (CD11b^+^Gr-1^+^) in the spleen. (**D**) MDSCs (CD11b^+^Gr-1^+^) in the liver. Data are presented as mean ± SD. ** *p* < 0.01 and *** *p* < 0.001 (Student’s *t*-test, *n* = 6 mice per group).

Previous experiments have confirmed the effect of GPR81 activation on T cells in AIH development. To further explore its impact on other immune cells, we assessed CD11c^+^ expression in the liver and spleen. Flow cytometry showed a significant decrease in CD11c^+^ expression in the 3,5-DHBA group, especially in the liver (Figure 3A,B). We also examined CD11b^+^Gr-1^+^ expression, which represents MDSC frequency. Flow data revealed an increase in double-positive cells in the 3,5-DHBA group (Figure 3C,D), indicating a potential role in the immune regulation of hepatitis. These results suggest that GPR81 activation affects T cell activation, reduces DC secretion, and promotes MDSC accumulation in Con A-induced hepatitis.

### 2.4. Inhibition of GPR81 Delays Malignant Progression of Hepatoblastoma Cells

**Figure 4:**
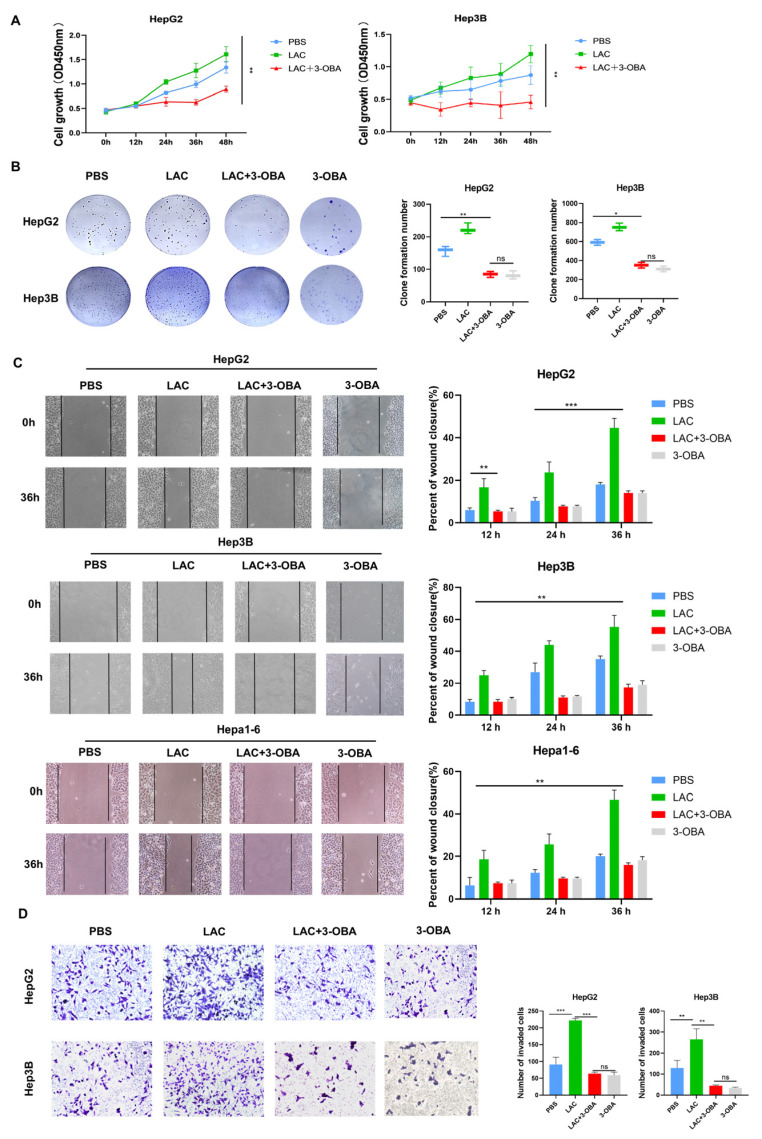
Inhibition of GPR81 slows HCC cells’ malignant progression. (**A**) Effect of lactate (5 mmol/L) and 3-OBA (5 mmol/L, GPR81 antagonist) on HepG2 and Hep3B cells’ proliferation for 0–48 h; data are presented as the average and SD. ** *p* < 0.01 (two-way ANOVA, Tukey’s post hoc test, *n* = 3 biological replicates). (**B**) Colony formation capacity for 14 days; data are presented as the average and SD. * *p* < 0.05, ** *p* < 0.01 (two-way ANOVA, Tukey’s post hoc test, *n* = 3 biological replicates). (**C**) Wound healing of HepG2, Hep3B, and Hepa1-6 cells over a 12–36 h period. Magnification: 100×. Data are presented as the mean ± SD. ** *p* < 0.01 and *** *p* < 0.001 (one-way ANOVA with Tukey’s post hoc test, *n* = 3 biological replicates). (**D**) Transwell cell invasion; Magnification: 100×. data are presented as the average and SD. ** *p* < 0.01 and *** *p* < 0.001 (two-way ANOVA, Tukey’s post hoc test, *n* = 3 biological replicates).

To investigate the role of GPR81 in HCC, HepG2 and Hep3B cells were treated with 5 mmol/L lactate, 5 mmol/L 3-OBA, or PBS. Functional assays showed that after 0–48 h, a noticeable divergence in absorbance at 450 nm appeared around 24 h. Specifically, the lactate-treated (LAC) group exhibited significant upregulation in cell proliferation, while the LAC+3-OBA group showed lower proliferation than both the LAC and the PBS group (Figure 4A). To further assess the impact of lactate on cell proliferation via GPR81, we cultured cells in six-well plates. After two weeks, colony formation was more robust in the LAC group; however, it was significantly inhibited in the LAC+3-OBA group. No significant difference was observed between the 3-OBA-only treatment group and the LAC+3-OBA group (Figure 4B). The Hepa1-6 cells were added to wound healing assays, and the experimental results revealed a marked increase in migration after lactate treatment but no significant increase in the 3-OBA group and LAC+3-OBA group (Figure 4C). Transwell invasion assays showed that lactate enhanced cell invasion, while GPR81 inhibition reduced this effect (Figure 4D). Notably, HepG2 cells were more sensitive to GPR81 regulation. These results suggest that lactate-mediated GPR81 activation promotes tumor cell proliferation.

### 2.5. GPR81 Inhibitors Show Similar Antitumor Effects in Hepa1-6 Cell Bearing Mice

**Figure 5:**
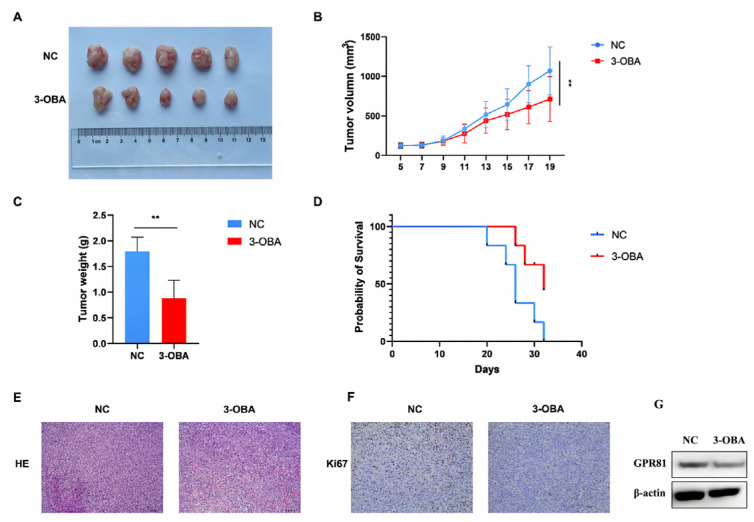
3-OBA showed a similar anti-tumor role in the Hepa1-6 tumor-bearing mice. (**A**) After modeling, the xenografts were removed and photographed. (**B**) Tumor volumes were documented every 2 days. Data are presented as the mean ± SD. ** *p* < 0.01 (Student’s *t*-test, *n* = 10 Hepa1-6 tumor-bearing mice per group). (**C**) Tumor weights were measured after 21 days of modeling (*n* = 10). (**D**) Survival of mice is shown as a Kaplan–Meier curve (*n* = 10), *p* < 0.01. (**E**) H&E staining of tumor tissue (scale bar 100 μm). (**F**) Representative IHC staining images of Ki67 in tumors, showing the malignant proliferation of the tumor (scale bar 40 μm). (**G**) Western blot analysis was performed to assess the differential expression of GPR81 in tumor-bearing tissues.

To investigate GPR81’s role in the tumor microenvironment, we conducted subcutaneous xenograft experiments using Hepa1-6 cells, followed by intraperitoneal injection of saline or 3-OBA. After 21 days, tumor grafts were excised, showing that tumors in the 3-OBA group were significantly smaller than those in the NC group (Figure 5A). Tumor length was measured every two days, and volume analysis (Figure 5B) revealed significantly suppressed growth in the 3-OBA group. Tumor weight was also significantly lower in the 3-OBA group (Figure 5C). Survival curves showed a higher survival rate in the 3-OBA group, with mice with tumors exceeding 3000 mm^3^ in size considered deceased (Figure 5D). H&E staining showed more necrosis in the 3-OBA group, indicating reduced tumor blood supply and slower growth (Figure 5E). Additionally, the 3-OBA group had fewer Ki67-positive cells, suggesting reduced HCC cell proliferation (Figure 5F). Western blot analysis was conducted to assess the protein levels of GPR81 in tumor tissues. The results showed that treatment with 3-OBA significantly decreased the protein levels of GPR81. These results demonstrate that the inhibition of GPR81 suppresses tumor growth.

### 2.6. Inhibition of GPR81 in HepG2 and Hep3B Cells, as Well as in Mouse Tumor Tissues, Leads to a Downregulation of PD-L1 Expression

**Figure 6:**
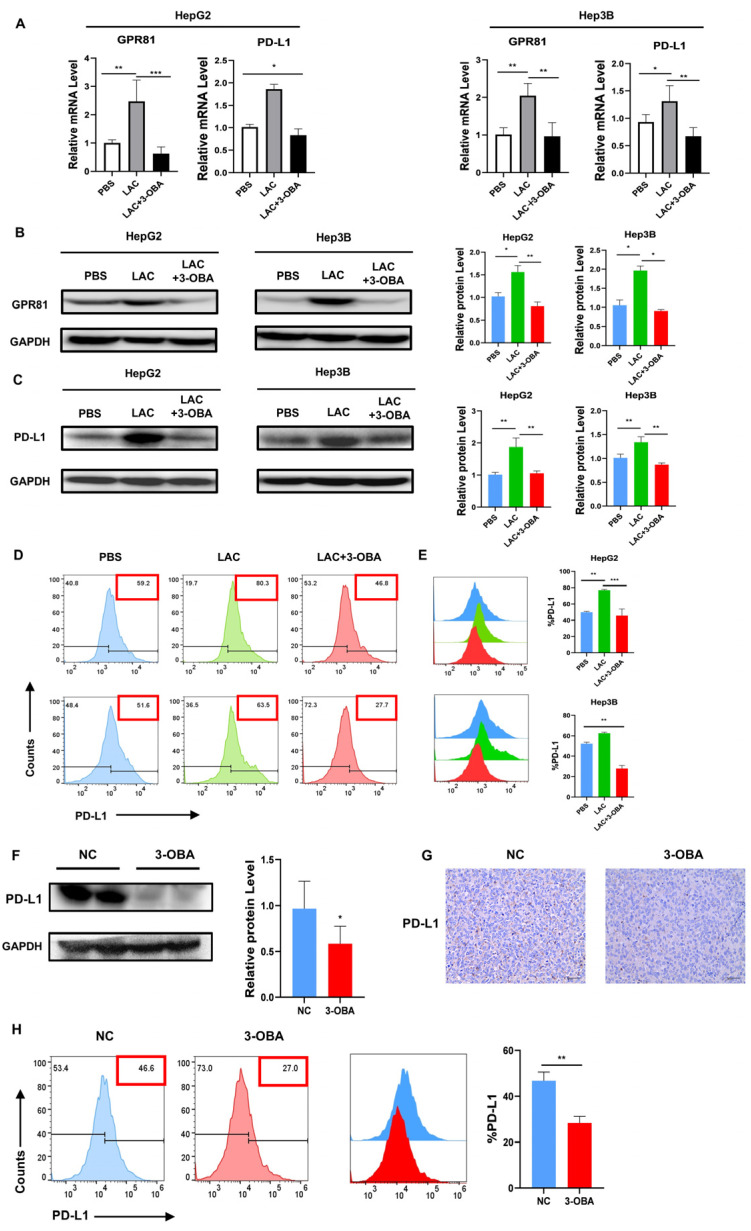
Inhibition of GPR81 in HCC and tumor tissues leads to the downregulation of PD-L1 expression. (**A**) The changes in the mRNA expression levels of GPR81 and PD-L1 in lactate- and 3-OBA-treated HepG2 and Hep3B cells. (**B**,**C**) Western blot results for the expression of GPR81 and PD-L1 at the protein level in HepG2 and Hep3B cells treated with lactate or 3-OBA. (**D**,**E**) Representative plots of PD-L1 in HepG2 and Hep3B examined using the flow cytometry assay. The data in (**A**–**E**) are presented as the average and SD. * *p* < 0.05, ** *p* < 0.01 and *** *p* < 0.001 (two-way ANOVA, Tukey’s post hoc test, *n* = 3 biological replicates). (**F**) Western blot results for PD-L1 expression at the protein level in tumor tissues. (**G**) Representative IHC staining images of PD-L1 in tumors (scale bar, 40 μm). (**H**) Tumor cell suspensions were collected, and PD-L1 expression in tumor tissues was measured by flow cytometry. The data in (**F**–**H**) are presented as the mean ± SD. * *p* < 0.05, ** *p* < 0.01 (Student’s *t*-test, *n* = 3 mice per group).

Lactate accumulation in tumors, a hallmark of the Warburg effect, regulates cancer cell metabolism and survival through the autocrine activation of GPR81. Brown et al. showed that lactate also promotes immune evasion by activating GPR81 on stromal dendritic cells in a paracrine manner [29]. Feng et al. demonstrated that GPR81 enhances lung cancer cells’ sensitivity to chemotherapy and counteracts immune evasion through the upregulation of checkpoint ligands like PD-L1 [32]. However, the expression pattern of GPR81 in HCC cells remains unclear. We investigated GPR81 expression in HepG2 and Hep3B cells using RT-PCR and Western blot. The results showed a significant reduction in GPR81 expression upon inhibition, leading to decreased mRNA and protein levels of PD-L1 (Figure 6A–C). Flow cytometry analysis confirmed a notable reduction in PD-L1 expression after GPR81 inhibition (Figure 6D,E). In vivo, the 3-OBA group exhibited reduced PD-L1 expression compared to the NC group (Figure 6F). Immunohistochemical staining further confirmed the presence of fewer PD-L1-positive cells in the 3-OBA group (Figure 6G). Additionally, flow cytometry of tumor tissue single-cell suspensions showed decreased PD-L1 expression upon GPR81 inhibition (Figure 6H). Thus, the inhibition of GPR81 suppresses tumor immune escape.

### 2.7. 3-OBA Remodels the Tumor Immune Microenvironment and Anti-Tumor Immune Response in Mice

**Figure 7:**
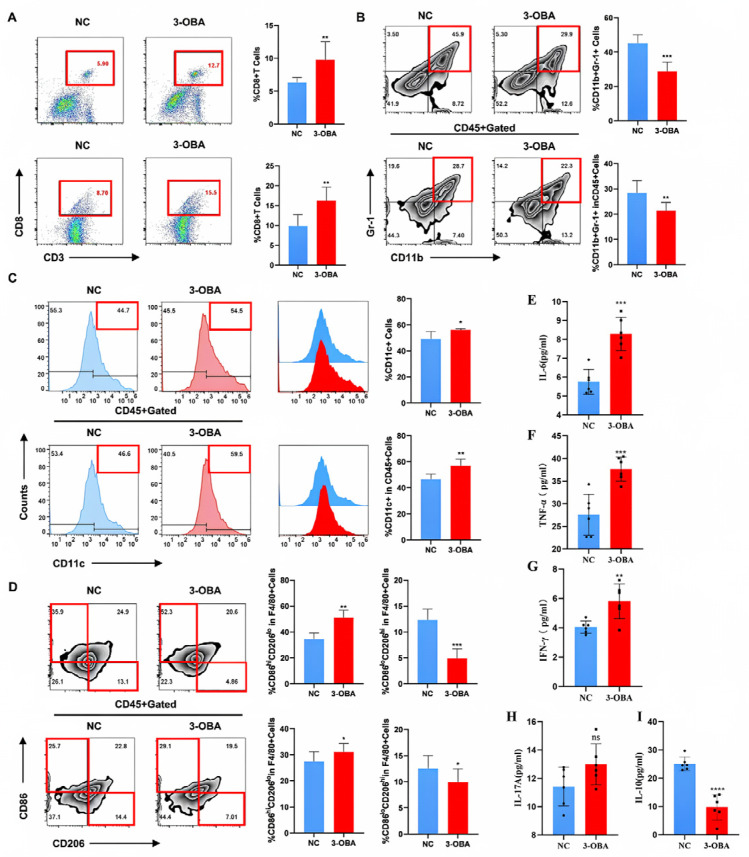
In vivo remodeling of the tumor immune microenvironment (TIME) and anti-tumor immune response induced by 3-OBA. Twenty-one days after orthotopic HCC implantation, tumor and spleen mononuclear cells were harvested and stained. Flow cytometry was applied to analyze the expressions of CTL (CD3^+^CD8^+^) (**A**), MDSCs (CD11b^+^Gr-1^+^) (**B**), DCs (CD11c^+^) (**C**), F4/80^+^CD86^hi^CD206^low^ M1, and F4/80^+^CD86^low^CD206^hi^ M2 (**D**). Flow cytometry was used to detect cytokines in tumor tissues, including IL-6 (**E**), TNF-α (**F**), IFN-γ (**G**), IL-17A (**H**), and IL-10 (**I**). Data are presented as the mean ± SD. * *p* < 0.05, ** *p* < 0.01, *** *p* < 0.001 and **** *p* <0.0001. (Student’s *t*-test, *n* = 6 mice per group).

The data above highlight the significant impact of GPR81 modulation on immune cell function, suggesting that inhibiting GPR81 activity could reshape the tumor immune microenvironment. We used flow cytometry to assess immune markers in the spleens and tumors of mice. In the 3-OBA group, the number of CD3^+^CD8^+^ T cells was significantly higher in both spleen and tumor tissues compared to the NC group (Figure 7A). Additionally, MDSCs (CD11b^+^Gr-1^+^) were significantly reduced in the spleen (Figure 7B), while overall DC (CD11c^+^) populations showed an increasing trend (Figure 7C). These changes align with an ideal tumor microenvironment. Further analysis revealed an increase in M1-type macrophages (CD86^hi^CD206^low^), known for their anti-tumor properties, and a decrease in M2-type macrophages (CD86^low^CD206^hi^), associated with tumor promotion (Figure 7D). Simultaneously, the concentrations of the cytokines IL-6, TNF-α, IFN-γ, and IL-17A were increased, while the concentration of IL-10 was decreased (Figure 7E–I). This is consistent with the anti-tumor pro-inflammatory effect observed following the inhibition of GPR81. These findings suggest that GPR81 could be a potential target for anti-tumor therapy.

## 3. Discussion

Cirrhosis is a major driving factor for hepatocellular carcinoma (HCC). A recent meta-analysis showed that, compared to other liver diseases, it nearly doubles the risk of HCC in patients with autoimmune hepatitis (AIH), increasing the likelihood of developing the disease from 0.19% to 0.53% [34]. Persistent cirrhosis and poor treatment outcomes are significant risk factors for the occurrence of HCC in AIH patients [35]. Furthermore, long-term immunosuppressive therapy in AIH patients increases the risk of extrahepatic malignancies compared to the general population [36]. Therefore, understanding the transition from inflammation to cancer and its molecular mechanisms is crucial for the early diagnosis and treatment of cancer. GPR81 is a G-protein-coupled receptor (GPCR) located on the cell membrane, and it is highly expressed in both inflammatory conditions and tumors, drawing considerable scholarly attention. However, its specific role in inflammation and tumors remains unclear.

Hoque R et al. first reported that GPR81 has immunosuppressive functions. Activation of this receptor in macrophages can alleviate liver and pancreatic injury models by downregulating the activity of the Toll-like receptor-mediated NLRP3 inflammasome and preventing the activation of NF-κB in macrophages [17]. In a glaucoma model, high expression of GPR81 in retinal microglial cells and astrocytes reduced the formation of the NLRP3 inflammasome [37]. Another study also supported the role of GPR81 as an immunosuppressant in the innate immune system. In vitro differentiated dendritic cells expressing GPR81 showed that both lactate and the non-metabolic GPR81 agonist CHBA inhibited MHC-II surface presentation and suppressed the spontaneous secretion of the pro-inflammatory cytokines IL-6 and IL-12, as well as TLR-induced secretion [29]. Administration of the stable GPR81 agonist 3,5-dihydroxybenzoic acid (3,5-DHBA) to pregnant mice alleviated endotoxin-induced uterine inflammation, preterm birth, and neonatal death [19]. Our findings also showed that treatment with 3,5-DHBA inhibited T cell activation and reduced dendritic cell (DC) secretion, improving AIH. This indicates that in AIH, GPR81 exerts anti-inflammatory effects by modulating immune cell activity.

GPR81 is highly expressed in cancer cells [38]. Therefore, the autocrine activation of GPR81 by lactate plays a key role in reprogramming cancer cell metabolism to adapt to the unique and harsh microenvironment of solid tumors, as well as in upregulating a series of common cancer cell defense mechanisms. In a 2020 study by Timothy P. Brown et al., GPR81 was found to be highly expressed in breast cancer. In a transgenic mouse model of breast cancer (MMTV-PyMT-Tg), the knockout of GPR81 resulted in the inhibition of breast cancer growth. Tumors from GPR81 knockout mice exhibited more tumor-infiltrating T cells and MHC-II high-expressing immune cells. Additionally, lactate induces the expression of programmed death ligand 1 (PD-L1) in tumor cells by activating GPR81 [29]. In triple-negative (TN) subtype breast cancer cells, the ratio of infiltrating CD8⁺ T cells to FOXP3⁺ T cells was significantly negatively correlated with GPR81 expression [39]. Beyond breast cancer, in lung cancer cells, GPR81 activation reduces intracellular cyclic adenosine monophosphate (cAMP) levels and inhibits protein kinase A (PKA) activity, leading to the activation of the transcriptional coactivator TAZ. The interaction between TAZ and the transcription factor TEAD is crucial for TAZ to activate PD-L1 expression [32]. In pancreatic cancer, hypoxia-inducible factor-1α (HIF-1α) inhibits T cell activity via the GPR81/mTOR/HIF-1α/STAT3 pathway [28]. However, the role of GPR81 in hepatocellular carcinoma remains unclear. In our study, we clearly demonstrated that inhibiting GPR81 activity significantly reduced PD-L1 expression. Furthermore, M1 macrophages, which are known for their anti-tumor properties, increased, while M2 macrophages, associated with tumor promotion, decreased. Concurrently, the concentrations of the cytokines IL-6, TNF-α, IFN-γ, and IL-17A increased, while the concentration of IL-10 decreased. These findings suggest that, in HCC, GPR81 aids tumor cell immune escape by upregulating PD-L1 expression and inhibiting T cell activity.

In summary, although the mechanisms of action of GPR81 in inflammation and tumors are similar, its functions and therapeutic implications differ between inflammation and tumor contexts. This suggests that GPR81 may play an important bridging role in the progression of inflammatory diseases to tumors. Therefore, in liver diseases, GPR81 could serve as a potential therapeutic target for cancer treatment. However, its application in inflammation should be approached with caution due to the complex interplay between inflammation and tumor progression.

## 4. Materials and Methods

### 4.1. Mice

Four- to six-week-old male C57BL/6 mice were purchased from Changsheng Bio-Technology Co., Ltd. (Lianyungang City, China) and housed in pathogen-free conditions with a 12 h light/dark cycle. Experiments began one week after adaptive feeding.

### 4.2. Cell Culture and Treatment

The human hepatocellular carcinoma cell lines HepG2 and Hep3B and the mouse hepatoma cell line Hepa1-6 were obtained from ATCC and maintained in our laboratory. Cells were cultured in Dulbecco’s modified Eagle medium (DMEM) (Gibco, Grand Island, NY, USA) supplemented with 10% fetal bovine serum (FBS) (Gibco, Grand Island, NY, USA) and 1% Penicillin-Streptomycin Solution (Beyotime, Beijing, China) at 37 °C with 5% CO_2_. L-Lactic Acid (purity > 90.0%) and 3-OBA (purity > 98.0%) were purchased from Aladdin (Shanghai, China).

### 4.3. Induction of the AIH

Mice were randomly assigned to two groups: the NC group (NC-AIH) and the 3,5-DHBA group (3,5-DHBA-AIH). AIH was induced by means of tail vein injection with Con A (10 mg/kg, Solarbio, Beijing, China). In the 3,5-DHBA group, mice were pretreated with 20 mg/kg 3,5-DHBA (APExBIO Technology LLC, Houston, TX, USA) via intraperitoneal injection 24 h before Con A treatment. The NC group received an equal volume of 0.9% normal saline. Serum, liver, and spleen tissues were collected 12 h after Con A administration. To assess survival, the concentration of Con A was adjusted to a lethal dose (20 mg/kg) [40,41], and mouse survival was monitored over a 48 h period following injection. The Kaplan–Meier method was employed to perform the survival analysis.

### 4.4. Xenograft Tumor Growth

A total of 1 × 10^6^ Hepa1-6 cells were subcutaneously injected into C57BL/6 mice. Tumor volumes were measured every other day using a digital caliper, with the formula V = ab^2^/2, where a is the long diameter and b is the short diameter. When tumor volumes reached 60–80 mm^3^, mice were randomly assigned to groups. The 3-OBA group received daily intraperitoneal injections of 3-OBA (50 mg/kg), while the NC group received an equal volume of 0.9% normal saline. Mice were sacrificed after 21 days of treatment for data collection.

### 4.5. ALT, AST, LDH and ALP Assessment

Serum levels of alanine aminotransferase (ALT), aspartate transaminase (AST), lactate dehydrogenase (LDH), and alkaline phosphatase (ALP) were measured using the Activity Assay Kit (Elabscience Biotechnology Co., Ltd., Wuhan, China) following the manufacturer’s instructions.

### 4.6. Hematoxylin and Eosin (H&E) Staining and Immunohistochemistry

Livers and tumors were harvested from model mice, immediately fixed in 4% paraformaldehyde, and embedded in paraffin. Tissue sections were prepared and stained with hematoxylin and eosin for histological analysis. Tumor sections were further stained for Ki67 (1:400, GB121141-100, Servicebio, Wuhan, China) and PD-L1 (1:400, 66248-1-Ig, Proteintech, Wuhan, China) for immunohistochemistry.

### 4.7. Flow Cytometry Analysis

Liver and spleen cells were isolated and stained with CD3, CD4, CD8, CD11b, CD11c, and Gr-1 antibodies (Biolegend, San Diego, CA, USA). For intracellular staining, cells were permeabilized with 0.2% Triton X-100 at 4 °C for 10–20 min, then stained with TNF-α, IL-17A, and IFN-γ antibodies (Biolegend, San Diego, CA, USA). HepG2 and Hep3B cells were treated with PBS, lactate (5 mmol/L), or 3-OBA (5 mmol/L) for 36 h, followed by PD-L1 antibody (Biolegend, San Diego, CA, USA) staining. Subcutaneous tumors were excised, minced, and digested with collagenase IV (1 mg/kg, Solarbio, Beijing, China), hyaluronidase (0.2 mg/kg, Solarbio, Beijing, China), and DNase I (0.2 mg/kg, Sigma-Aldrich, St. Louis, MO, USA) at 37 °C for 40 min. The digested tissues were filtered through a 70 µm filter, and tumor cells were stained with PD-L1 antibody (Biolegend, San Diego, CA, USA). Tumor and spleen cells were stained with CD3, CD8, CD11b, CD11c, F4/80, CD86, CD206, and Gr-1 antibodies (Biolegend, San Diego, CA, USA). Flow cytometry was performed for analysis.

### 4.8. Western Blot Analysis

HepG2 and Hep3B cells were treated with lactate (5 mmol/L) and 3-OBA (5 mmol/L) for 48 h, lysed with RIPA buffer, and proteins were collected and denatured. Then, they were electrophoretically treated and transferred onto a polyvinylidene difluoride (PVDF) membrane. The membrane was blocked with 5% non-fat milk for 2 h at room temperature, followed by incubation with primary antibodies against GPR81 (1:1000, DF2766, Affbiotech, Changzhou, Jiangsu Province, China) and PD-L1 (1:1000, 66248-1-Ig, Proteintech, China). Afterward, the membrane was incubated with the corresponding secondary antibody.

### 4.9. RNA Extraction and Real-Time PCR Analysis

Total RNA was extracted from HepG2 and Hep3B cells, and its quality was assessed using spectrophotometric analysis. Reverse transcription and PCR amplification were carried out using the RT-qPCR Kit (3735A, TAKARA, Kusatsu, Shiga Prefecture, Japan). The thermal cycling conditions were as follows: 5 min at 94 °C, followed by 30 cycles of 30 s at 94 °C, 30 s at 60 °C, and 60 s at 72 °C, with a final incubation at 72 °C for 5 min. Primers were synthesized by Sangon Biotech (Shanghai, China), and GAPDH was used as the internal reference for mRNA expression.

The primer sequences used are listed in the table below.

GeneForward Primers (5′-3′)Reverse Primers (5′-3′)
*GPR81*
CCGAGGAGGAACAGCGAAGCTGATGCCGTAGAGGAGCGATTG
*PD-L1*
GCCTCCACTCAATGCCTCAATTTGTTCACAACCACACTCACATGACAAG
*GAPDH*
AATCCCATCACCATCTTCCTTGAGGCTGTTGTCATACTTCT

### 4.10. CCK-8 Assay

HepG2 and Hep3B cells were seeded in 96-well plates with 4 × 10^3^ cells per well, respectively, until cell adhesion. Then, cells were treated with lactate (5 mmol/L) and 3-OBA (5 mmol/L) for 0–48 h. Subsequently, 10 µL of Cell Counting Kit-8 (CCK-8) reagent (Solarbio, Beijing, China) was added to each well, and the cells were incubated for 1 h at 37 °C. Absorbance at 450 nm was measured using a microplate reader (BioTek, Winooski, VT, USA).

### 4.11. Colony Formation Assay

HepG2 and Hep3B cell suspensions (8 × 10^3^ cells/mL, 100 µL) were seeded into each well of a 6-well plate and cultured in DMEM with 10% FBS. Cells were treated with lactate (5 mmol/L) and 3-OBA (5 mmol/L) for 12 days, with medium changes every two days. After treatment, cells were rinsed with PBS, fixed with 4% paraformaldehyde for 30 min, and stained with 0.1% crystal violet. Images were captured, and cell counts were performed using Image J (1.52p‌‌).

### 4.12. Wound Healing Assay

Four horizontal lines were drawn on the bottom of a six-well plate, and HepG2 and Hep3B cells were seeded until they reached 90% confluence. A 100 μL pipette tip was then used to scrape the monolayer cells perpendicular to the lines. Cell migration was recorded using an inverted microscope (CKX53, OLYMPUS, Tokyo, Japan) at 0, 12, 24, and 36 h after treatment with the lactate receptor inhibitor 3-OBA and lactate.

### 4.13. Transwell Invasion Assay

Logarithmically growing hepatoma cells were collected, washed three times with PBS, and resuspended in serum-free complete medium at a density of 1 × 10^5^ cells/mL. The cell suspension was added to the upper chamber of Transwell plates (Corning, New York, NY, USA), with 600 μL of DMEM containing 10% FBS in the lower chamber. After 36 h, non-migrated cells in the upper chamber were removed, and the migrated cells were fixed with 4% paraformaldehyde, stained with crystal violet, and washed with PBS. Migration was assessed by photographing five randomly selected fields using an inverted microscope.

### 4.14. Measurement of Cytokines

Tumor tissue from Hepa1-6-tumor-bearing mice was harvested and homogenized 21 days post tumor implantation, followed by sonication. After centrifugation at 13,000 rpm for 20 min, the supernatant was collected for cytokine concentration analysis. The concentrations of IL-6, TNF-α, IFN-γ, IL-10, and IL-17A were measured using the BD Cytometric Bead Array (CBA) Kit (BD Biosciences, San Diego, CA, USA), in strict accordance with the manufacturer’s instructions. Cytokine levels were determined by flow cytometry (Cytoflex LX, Beckman Coulter, Inc., Brea, CA, USA). Results are expressed as the average of measurements from duplicate wells. Data analysis was performed using CBA Analysis Software v1.1.15. The theoretical detection limits for the cytokines were as follows: IL-6—1.4 pg/mL; TNF-α—0.9 pg/mL; IFN-γ—0.5 pg/mL; IL-10—0.5 pg/mL; and IL-17A—0.8 pg/mL.

### 4.15. Statistical Analysis

Each experiment was repeated at least three times, and data are presented as means ± SD. Sample sizes were determined to ensure the validity of statistical analysis. Statistical significance between two groups was assessed using a two-tailed unpaired Student’s *t*-test, while differences among multiple groups were evaluated with one-way or two-way ANOVA, followed by Tukey’s post hoc test for pairwise comparisons. The comparison of survival curves was conducted by using log-rank test method. Exact *p*-values are reported in the figure legends, with *p* < 0.05 being considered statistically significant. Statistical analysis was performed using GraphPad Prism 7.0. Details of statistical tests and figure references are provided in the figure legends.

## Data Availability

Data is contained within the article.

## Abbreviations

The following abbreviations are used in this manuscript:

HCC	Hepatocellular carcinoma
AIH	Autoimmune hepatitis
3,5-DHBA	3,5-Dihydroxybenzoic acid
3-OBA	3-Hydroxybutyric acid
Con A	Concanavalin A
LAC	Lactate-treated
TME	Tumor microenvironment

## References

[1] Nault J.C., Paradis V., Ronot M., Zucman-Rossi J. (2022). Benign liver tumours: Understanding molecular physiology to adapt clinical management. Nat. Rev. Gastroenterol. Hepatol..

[2] Coussens L.M., Werb Z. (2002). Inflammation and cancer. Nature.

[3] Künzli M., Masopust D. (2023). CD4(^+^) T cell memory. Nat. Immunol..

[4] Weninger W., Manjunath N., von Andrian U.H. (2002). Migration and differentiation of CD8^+^ T cells. Immunol. Rev..

[5] Yin X., Chen S., Eisenbarth S.C. (2021). Dendritic Cell Regulation of T Helper Cells. Annu. Rev. Immunol..

[6] Locati M., Curtale G., Mantovani A. (2020). Diversity, Mechanisms, and Significance of Macrophage Plasticity. Annu. Rev. Pathol..

[7] Ivashkiv L.B. (2018). IFNγ: Signalling, epigenetics and roles in immunity, metabolism, disease and cancer immunotherapy. Nat. Rev. Immunol..

[8] Sanjabi S., Oh S.A., Li M.O. (2017). Regulation of the Immune Response by TGF-β: From Conception to Autoimmunity and Infection. Cold Spring Harb. Perspect. Biol..

[9] Mills K.H.G. (2023). IL-17 and IL-17-producing cells in protection versus pathology. Nat. Rev. Immunol..

[10] Afonina I.S., Cullen S.P., Martin S.J. (2010). Cytotoxic and non-cytotoxic roles of the CTL/NK protease granzyme B. Immunol. Rev..

[11] Richardson N., Wootton G.E., Bozward A.G., Oo Y.H. (2022). Challenges and opportunities in achieving effective regulatory T cell therapy in autoimmune liver disease. Semin. Immunopathol..

[12] Czaja A.J., Manns M.P. (2010). Advances in the diagnosis, pathogenesis, and management of autoimmune hepatitis. Gastroenterology.

[13] Harrington C., Krishnan S., Mack C.L., Cravedi P., Assis D.N., Levitsky J. (2022). Noninvasive biomarkers for the diagnosis and management of autoimmune hepatitis. Hepatology.

[14] Lohse A.W., Mieli-Vergani G. (2011). Autoimmune hepatitis. J. Hepatol..

[15] Ahmed K. (2011). Biological roles and therapeutic potential of hydroxy-carboxylic Acid receptors. Front. Endocrinol..

[16] Sun S., Li H., Chen J., Qian Q. (2017). Lactic Acid: No Longer an Inert and End-Product of Glycolysis. Physiology.

[17] Hoque R., Farooq A., Ghani A., Gorelick F., Mehal W.Z. (2014). Lactate reduces liver and pancreatic injury in Toll-like receptor- and inflammasome-mediated inflammation via GPR81-mediated suppression of innate immunity. Gastroenterology.

[18] Khatib-Massalha E., Bhattacharya S., Massalha H., Biram A., Golan K., Kollet O., Kumari A., Avemaria F., Petrovich-Kopitman E., Gur-Cohen S. (2020). Lactate released by inflammatory bone marrow neutrophils induces their mobilization via endothelial GPR81 signaling. Nat. Commun..

[19] Madaan A., Nadeau-Vallée M., Rivera J.C., Obari D., Hou X., Sierra E.M., Girard S., Olson D.M., Chemtob S. (2017). Lactate produced during labor modulates uterine inflammation via GPR81 (HCA(1)). Am. J. Obstet. Gynecol..

[20] Ranganathan P., Shanmugam A., Swafford D., Suryawanshi A., Bhattacharjee P., Hussein M.S., Koni P.A., Prasad P.D., Kurago Z.B., Thangaraju M. (2018). GPR81, a Cell-Surface Receptor for Lactate, Regulates Intestinal Homeostasis and Protects Mice from Experimental Colitis. J. Immunol..

[21] Wang X., Xie Z., Yuan J., Jin E., Lian W., Chang S., Sun G., Feng Z., Xu H., Du C. (2024). Sodium oligomannate disrupts the adherence of Ribhigh bacteria to gut epithelia to block SAA-triggered Th1 inflammation in 5XFAD transgenic mice. Cell Discov..

[22] Manoharan I., Prasad P.D., Thangaraju M., Manicassamy S. (2021). Lactate-Dependent Regulation of Immune Responses by Dendritic Cells and Macrophages. Front. Immunol..

[23] Raychaudhuri D., Bhattacharya R., Sinha B.P., Liu C.S.C., Ghosh A.R., Rahaman O., Bandopadhyay P., Sarif J., D’Rozario R., Paul S. (2019). Lactate Induces Pro-tumor Reprogramming in Intratumoral Plasmacytoid Dendritic Cells. Front. Immunol..

[24] Yang K., Xu J., Fan M., Tu F., Wang X., Ha T., Williams D.L., Li C. (2020). Lactate Suppresses Macrophage Pro-Inflammatory Response to LPS Stimulation by Inhibition of YAP and NF-κB Activation via GPR81-Mediated Signaling. Front. Immunol..

[25] Fan Q., Wu Y., Li M., An F., Yao L., Wang M., Wang X., Yuan J., Jiang K., Li W. (2021). Lactobacillus spp. create a protective micro-ecological environment through regulating the core fucosylation of vaginal epithelial cells against cervical cancer. Cell Death Dis..

[26] Ishihara S., Hata K., Hirose K., Okui T., Toyosawa S., Uzawa N., Nishimura R., Yoneda T. (2022). The lactate sensor GPR81 regulates glycolysis and tumor growth of breast cancer. Sci. Rep..

[27] Xie Q., Zhu Z., He Y., Zhang Z., Zhang Y., Wang Y., Luo J., Peng T., Cheng F., Gao J. (2020). A lactate-induced Snail/STAT3 pathway drives GPR81 expression in lung cancer cells. Biochim. Biophys. Acta Mol. Basis Dis..

[28] Yang X., Lu Y., Hang J., Zhang J., Zhang T., Huo Y., Liu J., Lai S., Luo D., Wang L. (2020). Lactate-Modulated Immunosuppression of Myeloid-Derived Suppressor Cells Contributes to the Radioresistance of Pancreatic Cancer. Cancer Immunol. Res..

[29] Brown T.P., Bhattacharjee P., Ramachandran S., Sivaprakasam S., Ristic B., Sikder M.O.F., Ganapathy V. (2020). The lactate receptor GPR81 promotes breast cancer growth via a paracrine mechanism involving antigen-presenting cells in the tumor microenvironment. Oncogene.

[30] Zhao Y., Li M., Yao X., Fei Y., Lin Z., Li Z., Cai K., Zhao Y., Luo Z. (2020). HCAR1/MCT1 Regulates Tumor Ferroptosis through the Lactate-Mediated AMPK-SCD1 Activity and Its Therapeutic Implications. Cell Rep..

[31] Liu X., Li S., Cui Q., Guo B., Ding W., Liu J., Quan L., Li X., Xie P., Jin L. (2024). Activation of GPR81 by lactate drives tumour-induced cachexia. Nat. Metab..

[32] Feng J., Yang H., Zhang Y., Wei H., Zhu Z., Zhu B., Yang M., Cao W., Wang L., Wu Z. (2017). Tumor cell-derived lactate induces TAZ-dependent upregulation of PD-L1 through GPR81 in human lung cancer cells. Oncogene.

[33] Yang K., Fan M., Wang X., Xu J., Wang Y., Tu F., Gill P.S., Ha T., Liu L., Williams D.L. (2022). Lactate promotes macrophage HMGB1 lactylation, acetylation, and exosomal release in polymicrobial sepsis. Cell Death Differ..

[34] Tarao K., Nozaki A., Ikeda T., Sato A., Komatsu H., Komatsu T., Taguri M., Tanaka K. (2019). Real impact of liver cirrhosis on the development of hepatocellular carcinoma in various liver diseases-meta-analytic assessment. Cancer Med..

[35] Mack C.L., Adams D., Assis D.N., Kerkar N., Manns M.P., Mayo M.J., Vierling J.M., Alsawas M., Murad M.H., Czaja A.J. (2020). Diagnosis and Management of Autoimmune Hepatitis in Adults and Children: 2019 Practice Guidance and Guidelines From the American Association for the Study of Liver Diseases. Hepatology.

[36] Hino-Arinaga T., Ide T., Kuromatsu R., Miyajima I., Ogata K., Kuwahara R., Hisamochi A., Torimura T., Sata M. (2012). Risk factors for hepatocellular carcinoma in Japanese patients with autoimmune hepatitis type 1. J. Gastroenterol..

[37] Harun-Or-Rashid M., Inman D.M. (2018). Reduced AMPK activation and increased HCAR activation drive anti-inflammatory response and neuroprotection in glaucoma. J. Neuroinflammation.

[38] Roland C.L., Arumugam T., Deng D., Liu S.H., Philip B., Gomez S., Burns W.R., Ramachandran V., Wang H., Cruz-Monserrate Z. (2014). Cell surface lactate receptor GPR81 is crucial for cancer cell survival. Cancer Res..

[39] Li X., Chen Y., Wang T., Liu Z., Yin G., Wang Z., Sui C., Zhu L., Chen W. (2023). GPR81-mediated reprogramming of glucose metabolism contributes to the immune landscape in breast cancer. Discov. Oncol..

[40] Gantner F., Leist M., Lohse A.W., Germann P.G., Tiegs G. (1995). Concanavalin A-induced T-cell-mediated hepatic injury in mice: The role of tumor necrosis factor. Hepatology.

[41] Ohta A., Sitkovsky M. (2001). Role of G-protein-coupled adenosine receptors in downregulation of inflammation and protection from tissue damage. Nature.

